# Loss function of *NtGA3ox1* delays flowering through impairing gibberellins metabolite synthesis in *Nicotiana tabacum*

**DOI:** 10.3389/fpls.2023.1340039

**Published:** 2023-12-15

**Authors:** Lele Deng, Chaofan Li, Qian Gao, Wenwu Yang, Jiarui Jiang, Jiaxin Xing, Haiying Xiang, Jun Zhao, Yekun Yang, Pengfei Leng

**Affiliations:** ^1^ Yunnan Key Laboratory of Tobacco Chemistry, R&D Center of China Tobacco Yunnan Industrial Co. Ltd., Kunming, Yunnan, China; ^2^ Crop Functional Genome Research Center, Biotechnology Research Institute, Chinese Academy of Agricultural Sciences, Beijing, China

**Keywords:** *Nicotiana tabacum*, flowering, gibberellins, genome editing, transcriptomic sequencing, metabolome sequencing

## Abstract

Flowering time, plays a crucial role in tobacco ecological adaptation besides its substantial influence on tobacco production and leaf quality. Meanwhile, it is sensitive to biotic or abiotic challenges. The plant hormones Gibberellins (GAs), controlling a number of metabolic processes, govern plants growth and development. In this study, we created a late flowering mutant *HG14* through knocking out *NtGA3ox1* by CRISPR/Cas9. It took around 13.0 and 12.1 days longer to budding and flowering compared to wild type Honghuadajinyuan. Nearly all of the evaluated agronomic characters deteriorated in *HG14*, showing slower growth and noticeably shorter and narrower leaves. We found that *NtGA3ox* was more prevalent in flowers through quantitative reverse transcription PCR analysis. Transcriptome profiling detected 4449, 2147, and 4567 differently expressed genes at the budding, flowering, and mature stages, respectively. The KEGG pathway enrichment analysis identified the plant-pathogen interaction, plant hormone signal transduction pathway, and MAPK signaling pathway are the major clusters controlled by *NtGA3ox1* throughout the budding and flowering stages. Together with the abovementioned signaling pathway, biosynthesis of monobactam, metabolism of carbon, pentose, starch, and sucrose were enriched at the mature stage. Interestingly, 108 up- and 73 down- regulated DEGs, impairing sugar metabolism, diterpenoid biosynthesis, linoleic and alpha-linolenic acid metabolism pathway, were continuously detected accompanied with the development of *HG14*. This was further evidenced by the decreasing content of GA metabolites such as GA4 and GA7, routine chemicals, alkaloids, amino acids, and organic acids Therefore, we discovered a novel tobacco flowering time gene *NtGA3ox1* and resolved its regulatory network, which will be beneficial to the improvement of tobacco varieties.

## 1 Introduction

Tobacco (*Nicotiana tabacum* L.) is one of the most globally planted industrial crops. L-nicotine, nornicotine, and other alkaloids could be extracted from tobacco, which have substantial therapeutic and medical uses (Papke et al., 2007; Harris et al., 2015). At the moment, excellent quality (flavor and aroma), acceptable production, and superior stress resistance are the prerequisites for tobacco cultivar. The flavor and aroma of tobacco leaves could be influenced by various chemical compounds including carbohydrates (starch, sugars, etc.), organic acids (neochlorogenic acid, hyoscypicrin, chlorogenic acid, cryptochlorogenic acid, etc.), phenols, and other substances (Yan et al., 2016). Tobacco production and quality potential can be elevated by increasing leaf number per plant, plant height, leaf size, and stem girth.

Flowering, a crucial stage during plants growth and development, marks the transition from vegetative to reproductive growth. Flowering time is a complex genetic variation which is also vulnerable to environmental factors, including photoperiod induction, nutrition distribution, temperature, and other abiotic stresses (McDaniel, 1996; Mattioli et al., 2008; Hackenberg et al., 2012; Song et al., 2013). Vernalization, temperature-sensitive, photoperiodic, and gibberellin pathways have been identified as the major approaches controlling plant flowering (Fornarua et al., 2010; Wellmer and Riechmann, 2010). The development, transmission, and interaction of internal signals monitor floral organs occurrence in response to environmental stimuli. Premature transition from vegetative to reproductive growth causes weaker plant growth, fewer and narrower leaves, all of which have an adverse effect on tobacco leaf yield and quality. It also causes increased energy consumption flow to the top floral organs, thereby decreased effective leaf number and insufficient nutrient uptake by the lower leaves. Early flowering also leads to chemical components’ imbalance in leaf, which affects leaf quality, is another concern of tobacco production (Kasperbauer, 1973). Therefore, manipulation on flowering time greatly matters tobacco production and development.

The most famous gene for flowering is *FT*, which was first identified in the late-flowering mutant of Arabidopsis (Turck et al., 2008). Since then, a large number of *FT* homologous genes have been discovered in rice, corn, wheat, soybean, and potato, etc. Overexpressing *AtTFL1* gene, a homolog of goldfish *CEN* gene, led to extended vegetative growth and delayed flowering in tobacco (Amaya et al., 1999). Phosphatidyl ethanolamine binding protein (PEBP), produced by the *FT* gene, stimulates plant flowering by circadian rhythmically binding to phosphatidylcholine (Liu et al., 2016; Khosa et al., 2021). The same as other plant species, tobacco flowering was also determined by genetic variations (McDaniel, 1996). However, due to relative lack of research progress in tobacco genome, only few flowering time genes have been cloned. Harig et al. (2012) identified four *FT* homologous genes in tobacco that belong to the PEBP family, among which only *NtFT4* was a flowering inducer, the other three were flowering inhibitors. *CET1-7*, a set of seven *TFL1* homologous genes, were found in tobacco (Xie et al, 2018). *In situ* hybridization analysis showed that *CET2* and *CET4* were only expressed in the axillary meristem, which was contrary to the prominent expression levels of *AtTFL1* and *CENs* in primary apical meristem during vegetative growth. Smykal et al. (2007) cloned MADS box gene members *SOC1* and *FUL* from tobacco. Both genes displayed comparable expression patterns in the day-neutral variety Hicks and the short-day variety Hicks MM under long-day or short-day conditions. In addition, overexpressing miRNA156 in cultivated tobacco led to delayed fluorescence (Lee et al., 2023). GAs have been frequently identified in a variety of plants (Binenbaum et al., 2018). They are broadly distributed in actively growing tissues, which promotes seed germination, stem elongation, and leaf expansion, as well as takes role in the control of plant flowering (Achard and Genschik, 2009; Davière and Achard, 2013; Hedden and Sponsel, 2015). GA promotes *FT* genes expression by repressing the DELLA protein (Porri et al., 2012), which mainly represses the expression of *SPL3* (squamosa promoter binding protein-like 3), the activating factor of FT (Galvão et al., 2012).

In this study, we created a late flowering mutant *HG14* through genome editing. In comparison to the wild type, both growth period and development were significantly hampered in the mutant, which was proved to be triggered by knocking out of *NtGA3ox1*. And *NtGA3ox1* transcriptome regulatory network, its impact on GA and chemical components were also investigated. Our study provides valuable breeding germplasm and gene resources for tobacco flowering biological breeding.

## 2 Materials and methods

### 2.1 Experimental materials and growth conditions

To investigate the role of *NtGA3ox1*, encoding a Gibberellin 3-beta-dioxygenase 1, in tobacco flowering, sgRNA (AATTACCCGAATCCCATGCATGG) was fused into the genome editing vector (pBUE-2gRNA-ZH) with T7E1 restriction sites (Xing et al., 2014). The recombinant vector was transformed into Agrobacterium *GV3101*, and genetic transformation of tobacco was performed in accordance with the leaf disc method (Gallois and Marinho, 1995). Next, CRISPR/Cas9-mediated *NtGA3ox1* knockout mutant was obtained, and the floral phenotypes and plant architecture were evaluated. A late-flowering mutant *HG14* was screened with significant delayed flowering and impaired plant development. Genomic DNA was extracted using the Plant Genomic DNA Kit (Tiangen Biotech, Beijing, China). Specific primer pairs (P-GSP5 + P-GSP3) were used to amplify sgRNA target sites and listed in **Supplementary Table 1**. After purification, PCR product was linked to pEASY-T1 vector (Transgen, Beijing, China). The mutation site was identified by Sanger sequencing.

The ecotype Honghuadajinyuan (Hongda) and *HG14* were floated in the greenhouse, with temperature at 26°C and the photoperiod was 16 h light/8 h darkness with 60% relative humidity. Tobacco seedlings with 35-40 days old were transplanted to the field. All the plants were field evaluated in Shilin (Yunnan province, 103°30’E, 24°81’N) in 2021 and 2022, and Xichang (Sichuan province, 102°06’E, 27°52’N) in 2022. The field experiment was conducted in a randomized block design, with 3 replicates, 2 rows in each plot, 60 plants for each replicate, with plant spacing of 0.5 m and row spacing of 1.2 m. Field management was carried out according to the field trial scheme.

### 2.2 Measurements of morphological characteristics regulated by *NtGA3ox1*

We next detailed measured the critical growth period, such as budding and flowering stage, and morphological characteristics of Hongda and *HG14*. Budding stage: the date when blossom buds of a plant were fully visible. Flowering stage: the date when 50% of the test plants bud emergence and flowering, respectively. Natural plant height: the distance from the ground-level base to the first fruit. Topping plant height: the distance from the ground-level base to the top of the stem. It was measured when removing the flower and the two guard leaves after flowering. Stem girth: the circumference between the fifth and sixth leaf during the first green fruiting stage. Leaf number: the number of leaves on the plant after removing the bottom leaves. Leaf length and width: the biggest leaf in the center was chosen. The width was determined by measuring the length vertically between the main vein and the widest section of the leaf surface. Leaf length was measured from the stem joint to the leaf tip on the front side of the leaf. For each trait, at least 15 plants from each replication were measured.

### 2.3 Cloning and phylogenetic analysis of *NtGA3ox1* gene

Research on structural domains is important for protein functional analysis. Gene-specific primers P-GSP5 and P-GSP3, developed based on the acquired cDNA fragment, were used to get the full-length sequence of the *NtGA3ox1*. The primers were listed in **Supplementary Table 1**. Then the amino acid sequence of *NtGA3ox1* was used as query for BLAST alignment with E-value < 0.1 in NCBI (http://blast.ncbi.nlm.nih.gov/Blast.cgi), and similar amino acid sequences were retrieved by BLASTP. The phylogenetic tree was built using the neighbor-joining method in MEGA version 7 (http://www.megasoftware.net/), with 1000 bootstrap replicates (Kumar et al., 2016).

### 2.4 *NtGA3ox1* expression pattern

There is close relationship between the tissue expression pattern of a gene and its function. The temporal and spatial expression pattern of *NtGA3ox1* were analyzed by qRT-PCR after *NtGA3ox1* was demonstrated to be the functional gene controlling *HG14* late flowering. The root, stem, leaf, and organ tissues from Hongda at the seedling, rosette, and flowering stages were harvested, respectively. Three individual plants were mixed as one replicate, three replicates for each. It was enveloped in foil film and frozen in liquid nitrogen immediately. Total RNA was extracted using TRIzol reagent (Invitrogen, Carlsbad, USA). RNA concentration and quality were determined using a Nanodrop 2000 instrument (Thermo, Waltham, USA). cDNA was synthesized by one-step gDNA Removal and cDNA Synthesis SuperMix (Genstar, Beijing, China) according to manufacturer’s instructions. qRT-PCR was performed on an ABI StepOnePlus instrument (Takara, Dalian, China) using an SYBR Premix Ex Teq II kit (Takara Bio., Kyoto, Japan). The RT-PCR program was as follows: melting at 95°C for 15 s, and amplification with 40 cycles of 95°C for 15 s and 60°C for 30 s. The relative expression level of the *NtGA3ox1* was analyzed according to the *2^­ΔΔCT^* method. *NtActin* was used as reference gene. The primer sequences were listed in **Supplementary Table 1**.

### 2.5 Transcriptomic sequencing analysis

The leaves of *HG14* and wild type Hongda at the budding, flowering, and mature stages were selected based on the spatial and developmental features of *NtGA3o*x*1* gene expression. Six replicates for each. RNA was isolated using the TRIzol reagent (Invitrogen, Carlsbad, USA), and their qualities were determined using the Nanodrop2000, respectively. cDNA libraries were constructed using the NEBNext^®^ Ultra™ RNA Library Preparation Kit for Illumina Sequencing System (NEB, USA). The transcriptome sequencing was conducted by Wuhan Huada Gene Technology Co. Ltd. Following the FastQC quality assessment, clean reads were generated and matched to the tobacco reference genome (ftp://ftp.solgenomics.net/genomes/Nicotiana_tabacum/, Edwards et al., 2017) using Bowtie 2 (v2.3.4.3).

Phyper (https://en.wikipedia.org/wiki/Hypergeometric_distribution) was utilized to perform GO (http://www.geneontology.org/) and KEGG (https://www.kegg.jp/) enrichment analysis based on hypergeometric testing, with a cutoff of Q-value ≤ 0.05. In order to further validate the transcriptome sequencing results, DEGs enriched in plant hormone signal transduction, plant-pathogen interaction, GA and amino acid biosynthesis were chosen for gene expression verification by qRT-PCR. qRT-PCR was conducted as previously described, with three independent biological replicates and three technical replicates, primers were designed by qPrimerDB (https://qprimerdb.biodb.org/, Lu et al., 2018) and listed in **Supplementary Table 1**. *NtActin* was employed as the reference gene.

### 2.6 Target metabolome sequencing analysis

For metabolome sequencing, the same samples for transcriptome analysis were employed. A freshly collected plant leaf was immediately frozen in liquid nitrogen and grounded into powder (30 Hz, 1 min). The leaf samples were weighed exactly at 50 mg and subsequently dissolved in 1 mL of MeOH/water/formic acid (15:4:1, v/v/v). After vortexing for 15 min, the mixture was centrifugated for 10 min (12000 r/min, and 4°C). The supernatant was transferred into sterile plastic microtubes and dried by evaporation. The residue re-suspended in 500 mL water containing 3.5% formic acid and 1 mL ethyl acetate, following by vortexing for 15 min, and centrifuged for 5 min (12000 r/min, and 4°C). 500 mL ethyl acetate was added to the re-extracted sample before the supernatant was mixed.

The combined ethyl acetate component was evaporation dried and then dissolved in ACN. 10 mL triethylamine and 10 mL 3-bromopropyltrimethylammonium bromide were added to the resultant solution. The reaction mixture was vortexed, heated at 90°C for 1 h, evaporated to dryness under nitrogen gas stream, redissolved in aqueous ammonia solution (90%), and then filtered through a 0.22 m membrane filter for further liquid chromatography mass spectrometry (LC-MS/MS) analysis. The targeted gibberellin metabolome sequencing was carried out by Metware biotechnology Co. Ltd. (Wuhan, China). A total of 18 substances including gibberellin A1 (GA1), GA15, and GA19, etc. (**Supplementary Table 2**) were examined, with four biological replications for each.

### 2.7 Chemical component analysis

Alkaloids, amino acids and other chemicals determine the economic value of tobacco leaf (Troje et al., 1997), which directly affects the cigarette quality. Based on the transcriptome profiling results, common DEGs enriched in Linoleic acid and fatty acid metabolism pathway. Therefore, alkaloids, chlorine, organic acids, amino acids, and total sugars in *HG14* and Hongda were analyzed to explore the effect of *NtGA3ox1* on chemical components. All samples were dried in an oven at 60°C for 30 min and then grounded thoroughly using a whirl wind grinding instrument. All testing procedures were in accordance with the Tobacco Industry Standard of People’s Republic of China. The content of routine chemical components of fresh tobacco leaves at the maturity stage were detected by a flow analyzer. The determination of routine chemicals, primary aromatic constituents, and inorganic nutrients were detected according to different analytical methods (**Table 1**), respectively. In this study, each line was tested with three independent biological repeats, with three plants for each.

**Table 1:** Analytical methods of chemical components in *HG14* and Hongda.

Chemicals	Analytical methods
Sugar (%)	Munson Walker
Chlorine (%)	Continuous flow
Total alkaloids (%)	Continuous flow
Volatile alkaloids (%)	Distillation
Potassium (%)	Flame photometry
Volatile organic acid (μg/g)	Ion Chromatography and Gas Chromatography-mass spectrometry
Free amino acids (μg/g)	Ion chromatography with integrated pulsed amperometric

### 2.8 Statistical analysis

Statistical significance between wild type Hongda and *HG14* was determined by one-way analysis of variance (ANOVA) or Student’s *t*-test in Excel. For DEG identification, a false discovery rate-adjusted *p* value (FDR) < 0.05 and fold change >4, DESeq2 (v1.4.5) was utilized. Significantly regulated metabolites between groups were considered at *P < 0.05 or **P < 0.01 by two-pairs Student’s *t*-test.

## 3 Results

### 3.1 Identification of delayed flowering mutant

**Figure 1:**
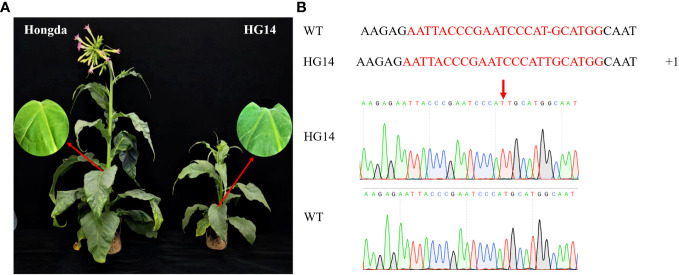
Phenotype **(A)** and genotype **(B)** of Hongda and late flowering mutant *HG14*.

GA regulates a number of metabolic pathways to govern plant growth and development. To investigate the potential roles of *NtGA3ox1* in tobacco growth and development, we generated *NtGA3ox1* knock-out mutants by CRISPR/Cas9. After comparison with the wild type Hongda, a late-flowering mutant *HG14* was identified, which exhibits significantly delayed flowering and stunting development, such as dramatically reduced biomass, shortened plant height, and increased glandular hairs (**Figure 1A**). According to the Sanger sequencing results, all clones had a T insertion in *NtGA3ox1* target site, gaining a pre-stop (**Figure 1B**). Stable transgenic lines were used for sub-sequent analysis.

### 3.2 Loss function of *NtGA3ox1* impaired plant development

**Figure 2:**
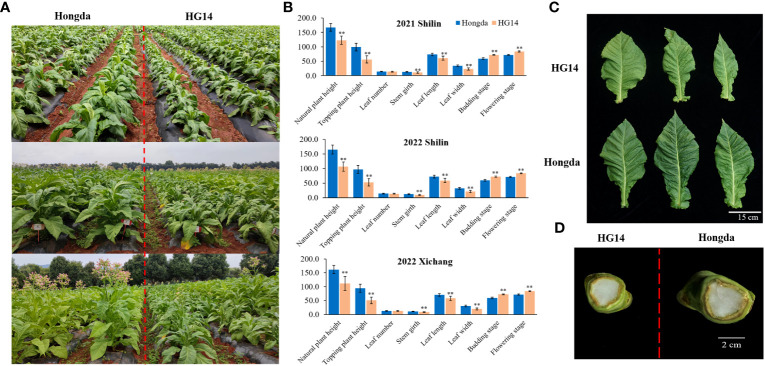
Growth and morphological characteristics of Hongda and *HG14* under field condition. **(A)** Growth variation of Hongda and *HG14* at multiple stages. **(B)** Statical analysis of morphological characteristics in Shilin (2021 and 2022) and Xichang (2022). **(C)** Leaf length and width. **(D)** Stem girth. Asterisks indicate significant differences between Hongda and *HG14* by Student’s *t*-test: *, *P* < 0.05; **, *P* < 0.01.

The agronomic characteristics of Hongda and *HG14* were further evaluated in 2021 and 2022 under field conditions. Similar results were obtained from Shilin in 2021 and Xichang 2022 (**Figure 2**). The development of *HG14* continuously lagged behind that of wild type Hongda, and the deficiency grew as development progressed (**Figure 2A**). Take the results of 2022 in Shilin as an example, *HG14* took 13.0 and 12.1 days longer than Hongda to reach the budding and flowering stages, respectively, at 72.4 and 83.8 days as opposed to 59.4 and 71.7 days (**Figure 2B**). *HG14* has shorter elliptical leaves, narrower stem and leaf angles, thin main veins, and lower growth potential. However, leaf number did not significantly alerted (**Figure 2B**). As an example, plant height of *HG14* was 106.4 cm, a reduction of 35.4% from Hongda’s (164.7 cm) in Shilin 2022 (**Figure 2B**). As for the topping plant height, it was much shorter for *HG14* at 52.5 cm compared to 96.9 cm for Hongda (*P* < 0.01). When compared to Hongda, *HG14* had significantly shorter and narrower leaves, averaging around 58.9 cm as opposed to 72.2 cm in length (*P* < 0.01), and 21.4 cm as compared to 32.4 cm in width (*P* < 0.01, **Figure 2C**). Additionally, stem girth of *HG14* was significantly smaller than Hongda, averaging 9.3 cm as compared to 12.2 cm (*P* < 0.01, **Figures 2B, D**). In summary, it can be predicted that *NtGA3ox1* should play a positive role in tobacco growth and development.

### 3.3 Structural domain and phylogenic analysis of *NtGA3ox1*

**Figure 3:**
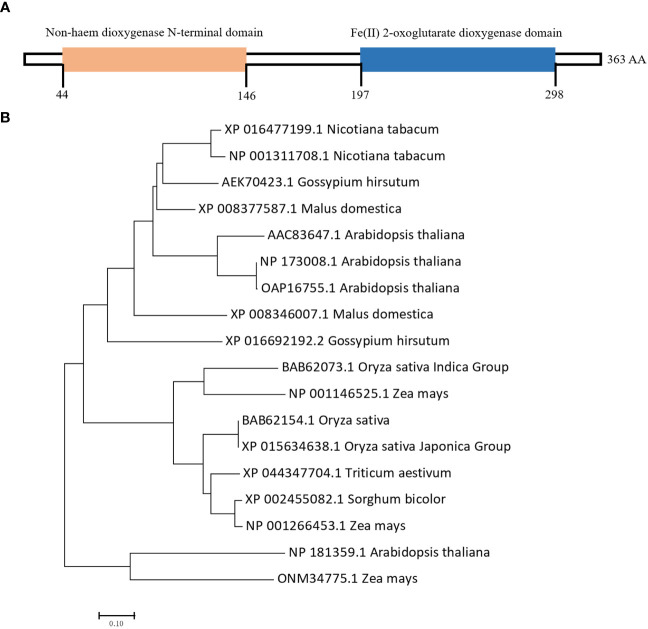
Domain and phylogenetic analysis of NtGA3ox1. **(A)** Functional domains of NtGA3ox1. **(B)** Phylogenic analysis of NtGA3ox1 among plant species.

We cloned the *NtGA3ox1* coding sequence from Hongda and searched the Pfam database (http://pfam.xfam.org) using the *NtGA3ox1* protein sequence. As shown in **Figure 3A**, NtGA3ox1 contains two functional domains, non-haem dioxygenase N-terminal domain (44-146, DIPR026992) and Fe(II) 2-oxoglutarate dioxygenase domain (197-298, PS51471). In plants, Fe(II) 2OG dioxygenase domain enzymes catalyze the formation of plant hormones, such as ethylene, gibberellins, etc.

Gibberellin dioxygenase is a large gene family in plants, governs the bio-synthesis of GA and crucial to the whole life cycle of plants. Therefore, phylogenetic tree was built utilizing *NtGA3ox1* and homologs of rice, Arabidopsis, wheat, and maize, etc. Gibberellin dioxygenase genes in plants exhibit evolutionary conservatism, as shown by their close relationship and sharing of two aforementioned dioxygenase domains. Interestingly, the phylo-analysis revealed that *Nicotiana tabacum*, *Arabidopsis*, and *Gossypium hirsutum* were grouped together, whereas *Oryza sativa* and *Zea maize* were clustered (**Figure 3B**), indicating the evolutionary distinctions between food and other crops.

### 3.4 Expression pattern of *NtGA3ox1*

**Figure 4:**
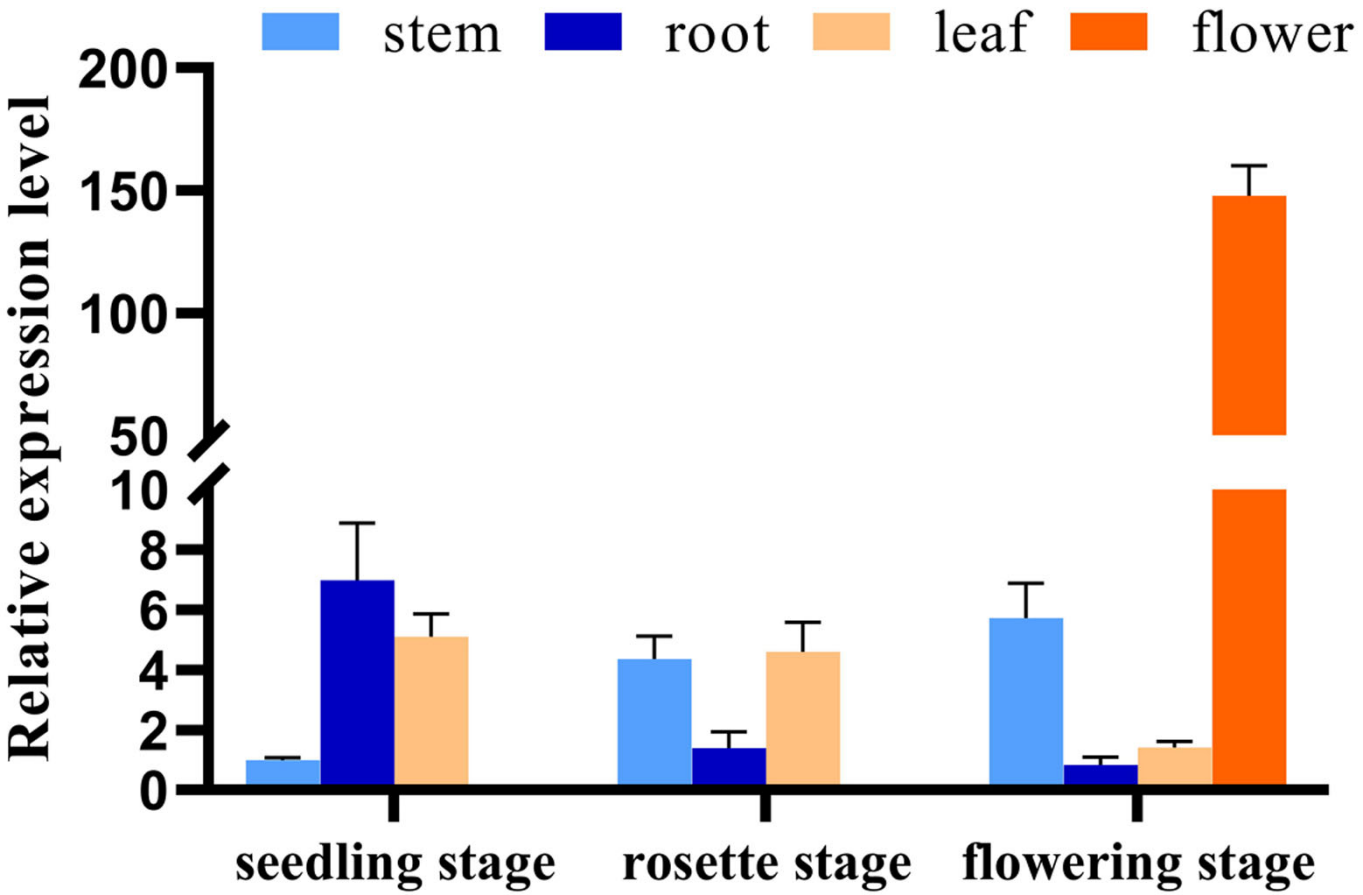
Temporal and spatial expression pattern of *NtGA3ox1* in Hongda.

We next investigated the expression pattern of *NtGA3ox1* across tissues and growth stages in Hongda. Lower expression levels were observed in stem compared to root and leaf during the seedling stage (**Figure 4**). Relatively higher expressions were detected in stem and leaf at the rosette stage. Flower has the highest expression level among the tested tissues, which further evidenced that *NtGA3ox1* involved in controlling plant flowering. The expression level in root gradually declined during the course of development, but it was dramatically raised in stem, which might control the flow from root to stem and thereby manipulate plant growth.

### 3.5 DEGs regulated by *NtGA3ox1*

36 cDNA libraries were created and sequenced to comprehensively assess the transcriptome alterations in Hongda and *HG14* at budding, flowering, and mature stages. All clean reads were compared and matched to tobacco reference genome v4.5 (https://solgenomics.net/organism/Nicotiana_tabacum/genome), with clean reads Q20 > 98.11% and Q30 > 94.07% (**Supplementary Table 3**), respectively. Pearson correlation analysis was used to assess the consistency of gene expression across samples, and high repeatability among biological replicates were observed for all time-points.

**Figure 5:**
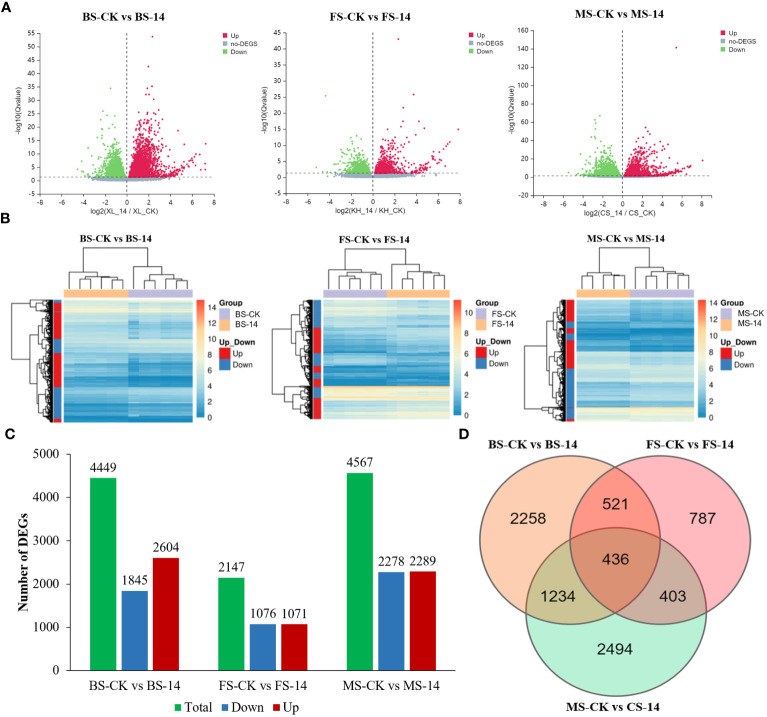
General analysis of transcriptome profiling in various developmental stages. **(A)** Volcano plot of differentially expressed genes (DEGs) between BS-14 vs. BS-CK, FS-14 vs. FS-CK, and MS-14 vs. MS-CK. **(B)** Pheatmaps for DEGs between BS-14 vs. BS-CK, FS-14 vs. FS-CK, and MS-14 vs. MS-CK. **(C)** Numbers of DEGs in the pair-wise comparisons. **(D)** Venn diagram of the number of DEGs revealed by pair-wise comparisons. BS, budding stage; FS, flowering stage; MS, maturing stage; CK, Hongda; 14, *HG14*.

Volcano maps were created to visualize the distribution of DEGs in each group. As shown in **Figure 5A**, the red, green, and grey dots indicate the up-regulated, down-regulated, and non-DEGs, respectively. A heatmap cluster analysis was conducted to illustrate the results of biological replicates, rows correspond to genes and columns to samples (**Figure 5B**). Those genes with similar expression patterns were grouped into four major clusters. Among the three stages, the smallest number of DEGs was observed in FS-14 (flowering stage-*HG14*) vs. FS-CK (flowering stage-Hongda) (2147), with 1071 up-regulated and 1076 down-regulated, respectively (**Figure 5C**). In contrast, the largest number of DEGs was found in MS-14 (mature stage-*HG14*) vs. MS-CK (mature stage-Hongda) (6202) including 2289 up-regulated and 2278 down-regulated. There were 4449 DEGs, including 2604 up-regulated and 1845 down-regulated, in BS-14 (budding stage-*HG14*) vs. BS-CK (budding stage-Hongda). Furthermore, FS-14 vs. FS-CK had the fewest DEGs in comparison to the other two groups, illustrated that although Hongda and *HG14* had distinct flowering times, most gene expression patterns were comparable because of their common genetic ancestry. The Venn diagram (**Figure 5D**) shows the overlap of DEGs among the three groups. Totally, 957 identical DEGs between BS and FS, 839 identical DEGs between MS and FS, and 1670 identical DEGs between MS and BS.

**Figure 6:**
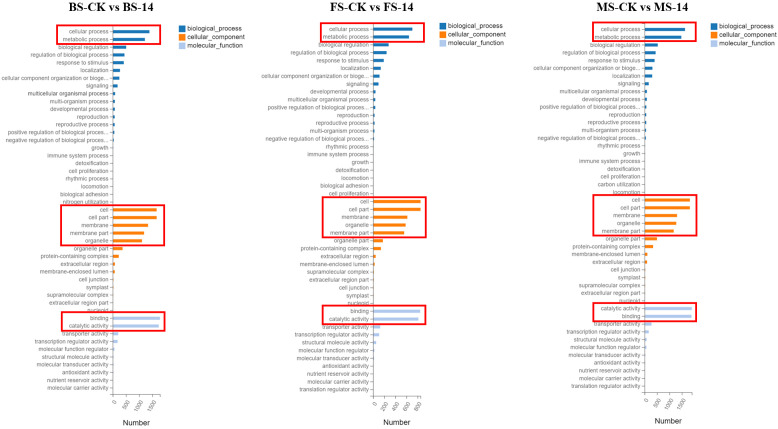
GO enrichment of the DEGs in three developmental stages.

**Figure 7:**
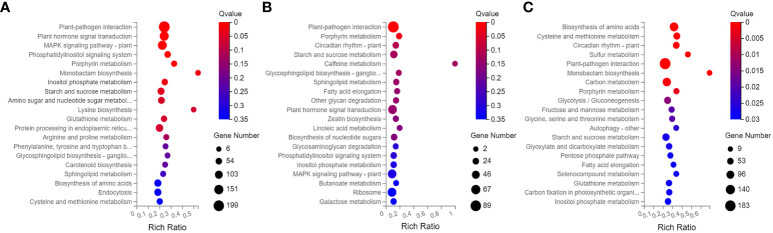
KEGG pathway enrichment of DEGs of the budding **(A)**, flowering **(B)**, and mature stage **(C)**.

The 4449 DEGs in BS-14 vs. BS-CK were classified into three functional groups by GO word enrichment analysis: biological process, cellular component, and molecular function (**Figure 6**). The two largest groupings in the biological process, as determined by the GO analysis, were “cellular process” and “metabolic process”. The DEGs were mostly engaged in cell, cell portion, membrane, etc. in the category of cellular component. “Binding” and “catalytic activity” genes predominated in the enrichment of molecular function. For the other two comparisons, MS-14 vs. MS-CK and FS-14 vs. FS-CK, the same was true. The KEGG pathway enrichment analysis revealed that a significant portion of DEGs for BS-14 vs. BS-CK group were mainly enriched in few pathways, including one plant-pathogen interaction pathway, three signaling pathways (plant hormone signal transduction, MAPK, and phosphatidylinositol), and porphyrin metabolism (**Figure 7**). Plant-pathogen interaction pathway and porphyrin metabolism were also discovered in the FS-14 vs. FS-CK group. When comparing the MS-14 vs. MS-CK group, those previously identified KEGG pathways were included. Other metabolism pathways, such as biosynthesis of monobactam, metabolism of carbon, fructose and sucrose, starch and sucrose, were also enriched.

**Figure 8:**
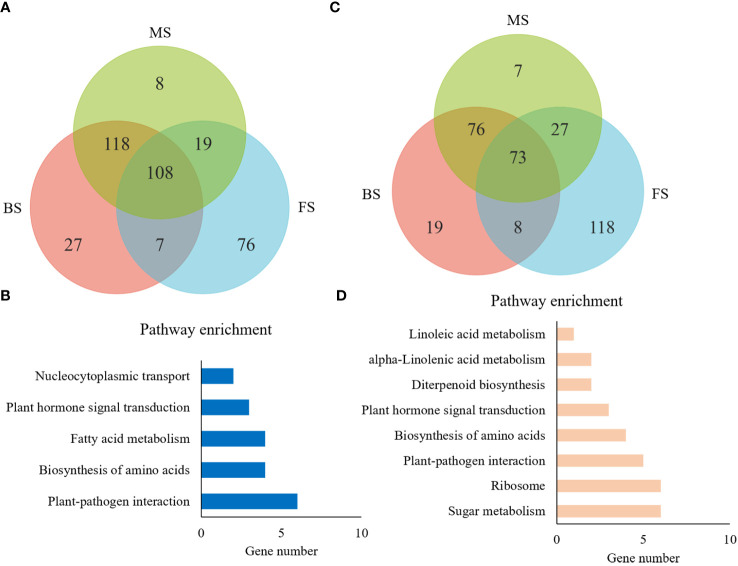
General analysis of 436 common DEGs across multiple stages. **(A, C)** Venn diagram of the number of common up and down resulted genes revealed by pair-wise comparisons, respectively. **(B, D)**, KEGG pathway enrichment for common up and down regulated genes, respectively. BS, budding stage; FS, flowering stage; MS, maturing stage; CK, Hongda; 14, *HG14*.

Totally, 436 comparable DEGs were identified among the three groups, implying that they play a continuous role throughout *NtGA3ox1*-regulated tobacco leaf development. 108 DEGs were up-regulated with loss function of *NtGA3ox1* in all three stages (**Figure 8A**), and 30 DEGs were found to be enriched in known KEGG pathways (**Figure 8B**) - diterpenoid biosynthesis (KO00904), plant-pathogen interaction (KO04626), ribosome (KO03010), and plant hormone signal transduction (KO04075). Furthermore, 73 down-regulated DEGs were obtained in *HG14* and mostly enriched in KEGG pathways, including those for plant-pathogen interaction and plant hormone signal transduction (**Figures 8C, D**), which corresponded to the up-regulated DEGs and these show their diverse roles in plant growth and associations with Gibberellin metabolism.

**Figure 9:**
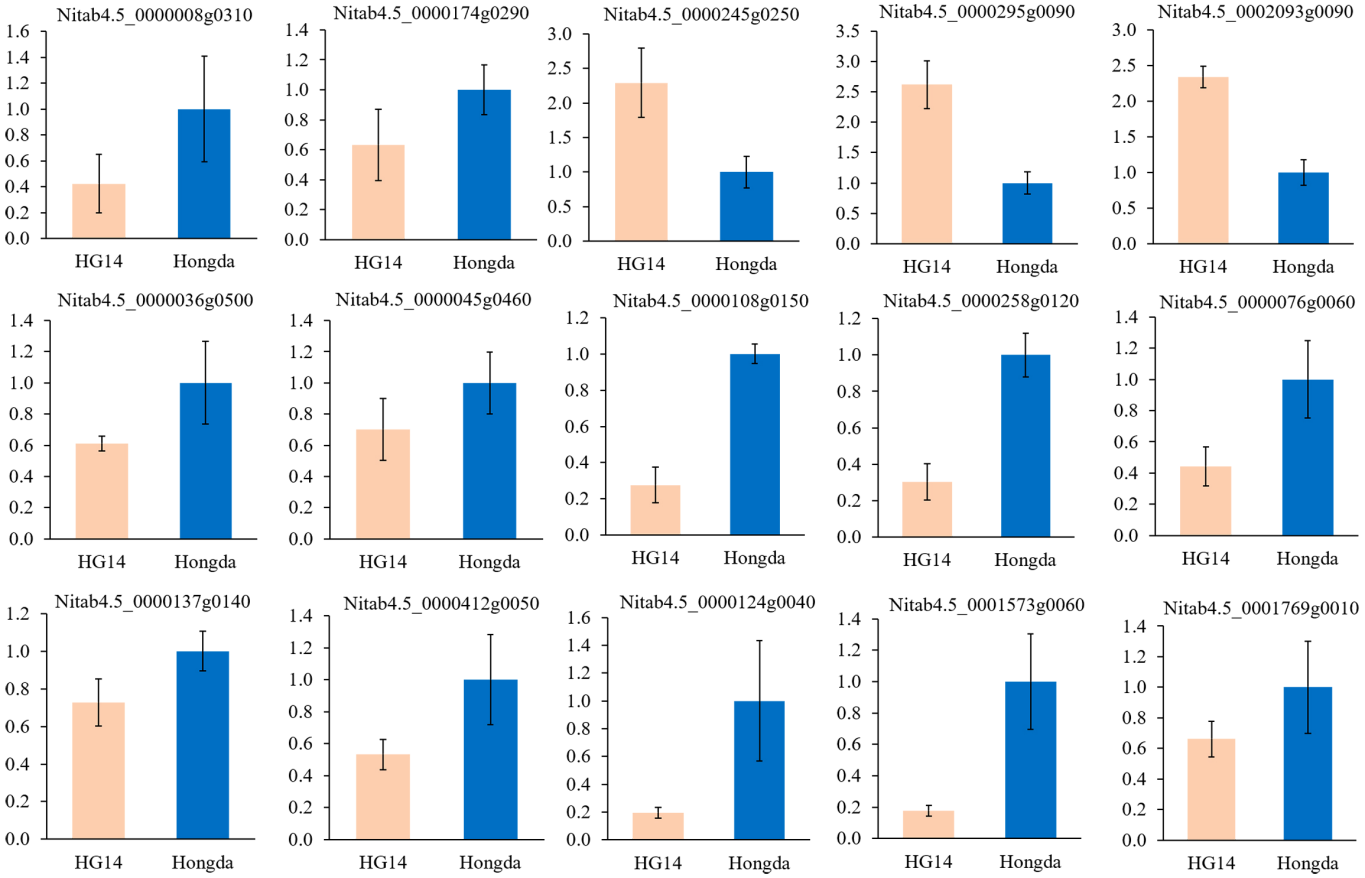
Verification of plant hormone signal transduction, plant-pathogen interaction, GA and amino acid biosynthesis by qRT-PCR. Relative expression levels of candidate genes were calculated using the actin gene as a standard. Error bars represent the standard deviation of six replicates.

We further selected 5 genes in plant hormone signal transduction (*Nitab4.5_0000008g0310*, *Nitab4.5_0000174g0290*, *Nitab4.5_0000245g0250*, *Nitab4.5_0000295g0090*, and *Nitab4.5_0002093g0090*), 4 genes in plant-pathogen interaction pathway (*Nitab4.5_0000036g0500*, *Nitab4.5_0000045g0460*, *Nitab4.5_0000108g0150*, and *Nitab4.5_0000258g0120*), 3 genes in GA metabolism (*Nitab4.5_0000076g0060*, *Nitab4.5_0000124g0040*, and *Nitab4.5_0001573g0060*), 2 genes in amino acid biosynthesis pathway (*Nitab4.5_0000412g0050* and *Nitab4.5_0001769g0010*), and 1 gene in organic acid metabolism pathway (*Nitab4.5_0000137g0140*) to verify the transcriptome sequencing results at the flowering stage. FPKM for these genes across the three time periods were presented in **Supplementary Figure 1**, and the differential fold changes ranging from 0.09 to 68.76. The qRT-PCR results suggested that most genes were down regulated and showed consistent expression pattern in Hongda and *HG14* with knock out of *NtGA3ox1* (**Figure 9**).

### 3.6 Loss function of *NtGA3ox1* impairs gibberellin metabolic

**Figure 10:**
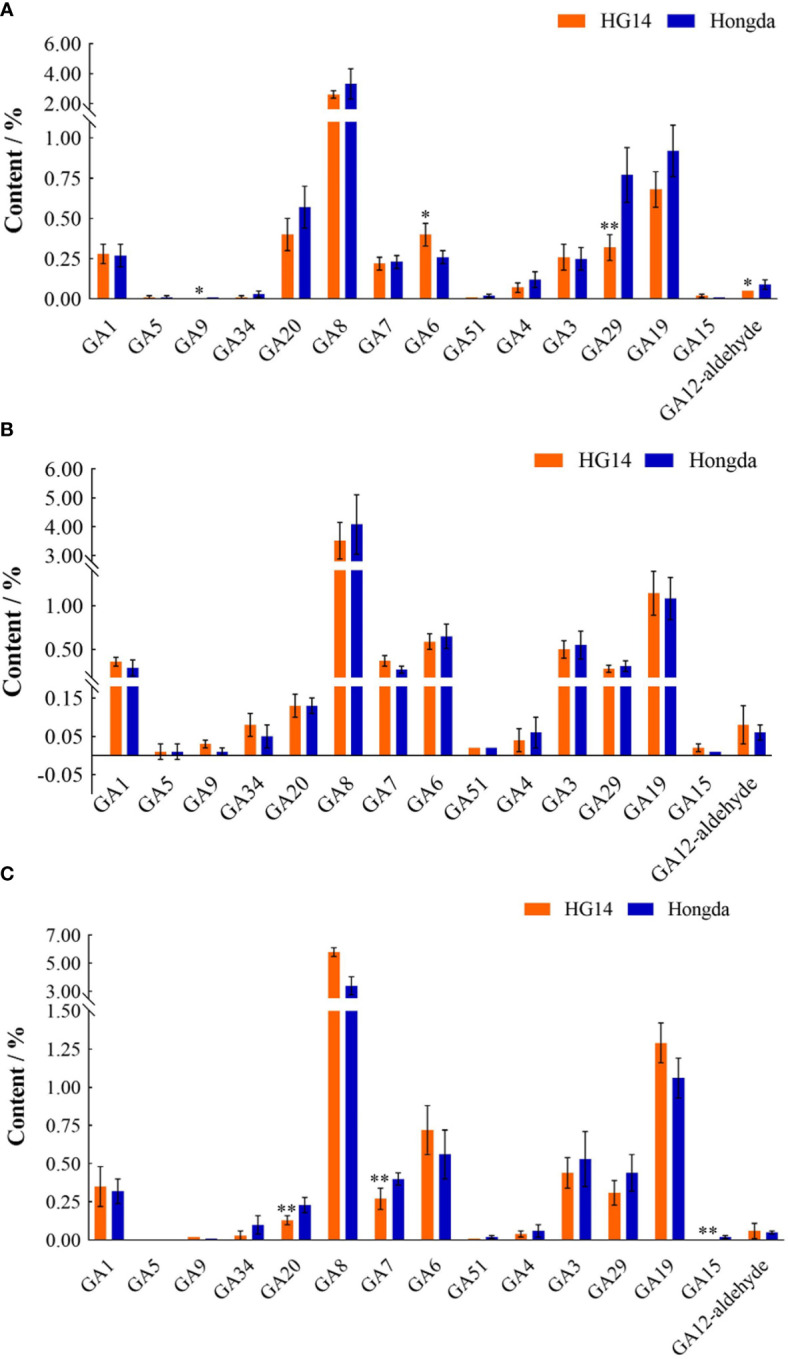
Gibberellin metabolites contents in Hongda and HG14 at the budding **(A)**, flowering **(B)**, and mature stage **(C)** by metabolome sequencing. Asterisks indicate significant differences between Hongda and *HG14* by Student’s *t*-test: *, *P* < 0.05; **, *P* < 0.01.

We focused on the gibberellin metabolites accumulation variation in Hongda and *HG14* throughout various leaf development stages. At the budding and flowering stages, the overall content of the 15 detected gibberellins in *HG14* was much lower than that in Hongda, whereas greater at the mature stage as a result of the large rise in GA8 in *HG14* (**Figure 10**). As shown in **Figure 10A**, knock out of *NtGA3ox1* resulted in a substantial reduction in the levels of three GA metabolites, including a 50.22% reduction in GA12-aldehyde (*P* < 0.05), a 58.91% reduction in GA9 (*P* < 0.05), and a 58.39% reduction in GA29 (*P* < 0.01). And a substantial 53.91% rise in GA6 (*P* < 0.05) was also observed. Only GA9, transiently elevated at 180.62%, was discovered in the metabolic profiles of GAs from the two genotypes at the flowering stage (**Figure 10B**). As shown in **Figure 10C**, three GAs metabolites, GA20, GA7, and GA15, were found to be highly down-regulated at the mature stage, with a decrease of 42.56%, 33.05%, and 71.49%, respectively. Additionally, knocking out *NtGA3ox1* caused the downregulation of GA51, GA4 and GA29 as well as leaf aging.

### 3.7 Chemical contents effected by NtGA3ox1

#### 3.7.1 Routine chemicals

**Figure 11:**
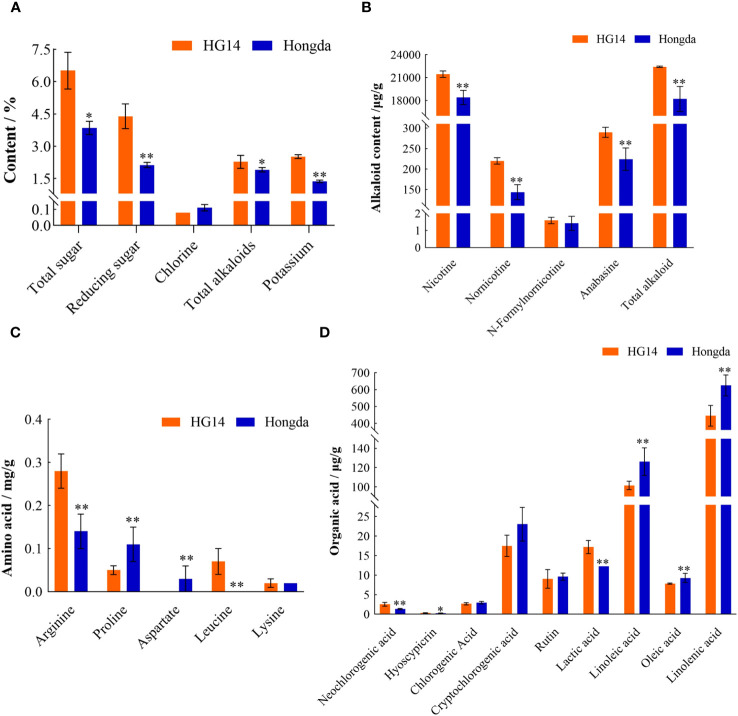
Chemical contents in Hongda and HG14 after harvesting. **(A)** Routine chemicals. **(B)** Alkaloids. **(C)** Amino acids. **(D)** Organic acids. Asterisks indicate significant differences between Hongda and *HG14* by Student’s *t*-test: *, *P* < 0.05; **, *P* < 0.01.

The chemical contents of *HG14* and Hongda were shown in **Figure 11A**. Basically, all examined chemicals in *HG14* except chlorine were dramatically elevated due to the loss function of *NtGA3ox1*. For instance, there was an increase of 69.18% of total sugar in *HG14* (6.51%) compared to Hongda (3.85%). Reducing sugar in the *HG14* was increased by 106.26% compared to Hongda, 4.39% vs. 2.13%. Furthermore, there was an 83.94% rise in potassium and a 19.41% increase in total alkaloids. Surprisingly, *HG14* has a 32.35% lower level of chlorine than wild type Hongda.

#### 3.7.2 Alkaloids

Around 95% of the tobacco alkaloids are nicotine, and the other 5% are minor alkaloids including nornicotine, anabasine, and nicotyrine, etc. (Shen and Shao, 2006). **Figure 11B** exhibits alkaloid contents difference in *HG14* and Hongda. Nicotine level in *HG14* was 21460.36 μg/g, much higher than in wild type Hongda which was 18105.14 μg/g. Accordingly, the total alkaloids in *HG14* and Hongda were 22425.96 μg/g and 18206.99 μg/g, respectively, with a substantial increase of 23.17%. Additionally, compared to the wild type, loss function of *NtGA3ox1* significantly increased the minor Alkaloids content in *HG14*, such as 53.10%, 77.97%, and 29.11% higher contents of Nornicotine, N-Formylnornicotine, and Anabasine, respectively (**Figure 11B**).

#### 3.7.3 Amino acids

To verify whether *NtGA3ox1* has an impact on the synthesis of amino acids, the contents of Arginine, Proline, Aspartate, Leucine, and Lysine were tested in Hongda and *HG14*. Among the five tested amino acids, arginine showed a notable 95.35% increase, rising from 0.14 mg/g in wild type Hongda to 0.28 mg/g in *HG14* (**Figure 11C**). Leucine was another amino acid that was significantly elevated by the lost function of *NtGA3ox1*, 0.07 mg/g in *HG14*, while it wasn’t detected in Hongda. Proline contents was 0.11 mg/g in Hongda, and 0.05 mg/g in *HG14*, decreasing by 60.3%. Aspartate, greatly decreased without *NtGA3ox1*, was not detected in *HG14* whereas wild type had an aspartate content of 0.03 mg/g.

#### 3.7.4 Organic acids

The contents of Neochlorogenic acid, Hyoscypicrin, Chlorogenic Acid, Cryptochlorogenic Acid, and Rutin in Hongda and *HG14* were measured to further support the involvement of *NtGA3ox1* in controlling the synthesis of organic acids in tobacco. Neochlorogenic acid and hyoscypicrin concentrations in Hongda were 1.34 mg/g and 0.25 mg/g, respectively, whereas they were 2.56 mg/g and 0.30 mg/g in *HG14*, which were 91.52% and 20.27% higher than those in Hongda, respectively (**Figure 11D**). The content of chlorogenic acid, cryptochlorogenic acid, and rutin were 2.98 mg/g, 23.03 mg/g, and 9.63 mg/g in Hongda, while they were 2.68 mg/g, 17.51 mg/g, and 9.05 mg/g, decreasing by 10.08%, 23.98%, and 6.02% in *HG14*, respectively (**Figure 11D**). Linoleic acid and Linolenic acid contents in Hongda were 126.25 mg/g and 624.96 mg/g, whereas they were 101.58 mg/g and 444.84 mg/g in *HG14*, decreased by 19.54% and 28.82%, correspondingly (**Figure 11D**).

## 4 Discussion

The *NtGA3ox1* family genes involved in various stages of plant growth and development. In this study, we identified that the knockout of *NtGA3ox1* by CRISPR/Cas9 caused later flowering and stunted development and growth in tobacco plants (**Figure 1**). The late flowering mutant *HG14* was considerably shorter than WT, the reason of which might be that that increased gibberellin promotes the transition of tobacco from vegetative development to reproductive growth (Binenbaum et al., 2018). Stem and root elongation were regulated by gibberellin synthesis genes. Inhibiting *GA3ox* expression in plants can lead to dwarfing traits, while modifying *GA3ox* expression can alter the plant growth. In contrast to wild type, dwarf mutant plants were produced when *StGA3ox2* expression was knocked down, which affects node spacing (Roumeliotis et al., 2013). A structural domain loss in *OsGA3ox2*, encoding a rice stalk height association protein, caused various degrees of dwarfing characteristics which resulted from the influence on the production of active GA (Itoh et al., 2001; Sakamoto et al., 2004). Additionally, using American tobacco, common tobacco, and wood tobacco, we created the phylogenetic tree for *NtGA3ox1* in tobacco (**Supplementary Figure 2**). Three major clades could be divided - flavonoid metabolic pathway, predicted feruloyl CoA ortho-hydroxylase, and gibberellin metabolic pathway. *NtGA3ox1* was most closely related to the GAs among tobacco, followed by feruloyl CoA ortho-hydroxylase and flavonol synthase (**Supplementary Figure 2**). Flavonoid metabolic pathway is highly conserved across the flowering plants (Grotewold, 2006; Tanaka et al., 2008), and are essential for the whole biological processes (Hu et al., 2020; Shen et al., 2022). These all imply that *NtGA3oxs* might play various roles in tobacco growth and development.

*NtGA3ox1* was found to be highly expressed in root and leaf at the early developmental stages and drastically increased in the stem along with plant growth (**Figure 4**). This might be attributed to the large amount of active GAs requirement in the early-developed stalk to meet the growth demand. *GA3oxs* expression pattern differs in plant species. It was reported that higher *GA3ox* levels were detected in stem tips, flower buds and roots, while almost no expression in leaves in tobacco (Itoh et al., 1999). Our findings, however, revealed that relative greater *NtGA3ox1* expression existed in the leaf during seedling and rosette stages (**Figure 4**). Two rice *OsGA3ox* genes were differential expressed among tissues, with *OsGA3ox1* being more prevalent in unopened flowers and *OsGA3ox2* in expanded leaves (Itoh et al., 2001). Four *AtGA3ox* genes were active at multiple developmental stages in Arabidopsis. *AtGA3ox3* and *AtGA3ox4* were mostly expressed in flowers and fruit pods, while *AtGA3ox1* and *AtGA3ox2* were highly expressed in germinating seeds and stem tips (Mitchum et al., 2006).

Although GA metabolites is known to control flowering in plants, the regulatory network of *NtGA3ox1* knockdown in tobacco is less understood. The biggest number of 4449 DEGs was identified at the budding stage, in line with higher *NtGA3ox1* levels in younger leaves, which showed rapid transcriptional reprogramming. The flowering stage had the fewest DEGs among the three groups, this might be attributed to the relative lower *NtGA3ox1* level in leaves. It was noted that out of the 436 common DEGs across the three time periods, 108 and 73 DEGs exhibited continuous upregulation and downregulation, respectively, and 255 displayed inconsistent expression patterns. In addition to *NtGA3ox*1 identified in this study, other gene/s may also have regulatory effects on the expression of these DEGs. As seen from **Figure 8**, budding and mature stage shared most DEGs with the same expression pattern, while flowering stage has the largest amount of specific DEGs, suggesting their opposite effects between vegetative growth to reproductive growth.

It is worth noting that *NtGA3ox1* triggered multiple genes in the plant-pathogen interaction pathway (KO04626). The glandular trichomes, which were epidermal cell projections found in most vascular plants, can protect the plant against insects, herbivores, and water loss (Traw and Bergelson, 2003; Olsson et al., 2009). Gibberellins were involved in the formation of Arabidopsis glandular trichomes (Chien and Sussex, 1996; Perazza et al., 1998). Previously, C2H2 zinc-finger transcription factors (GIS) was proved to be component of GL3/EGL3-GL1-TTG1 transcriptional activator complex to regulate trichome initiation through GA responding in Arabidopsis and tobacco, thereby increase the content of tobacco nicotine (Gan et al., 2006; Liu et al., 2017). This could be attributed to the higher *AtGIS* expression in trichomes, and thereby triggers excess glandular trichomes differentiation in tobacco. By far, no association has been found between the formation of glandular trichomes and *NtGA3ox1* in tobacco. Further research on the expression level of *NtGA3ox1* in glandular trichomes might provide an answer to its corresponding function in glandular trichomes formation. In addition to providing leaf protection, tobacco trichomes secretion is closely associated with tobacco aroma release, such as diterpenoids (Wagner, 1991). As a result, we looked into the endogenous chemical components of *HG14*. Organic acids such as linoleic acid, oleic acid, and linolenic acid were significantly reduced in *HG14*, which may be related to the decrease of internal GA content. Additionally, *HG14* had a considerable accumulation of nicotine and other alkaloids, which might be correlated with the appearance of glandular trichomes, however, they might be non-secretory. Taken together, *NtGA3ox1* could be a target gene for improving tobacco flavor and resistance to environmental hazards when combined with the fact that *HG14* carries more glandular trichomes (**Figure 1A**) and impaired gibberellin metabolites (**Figure 10**).

Furthermore, *Nitab4.5_0003156g0100* expression in the linoleic acid metabolism pathway (KO00591) and *Nitab4.5_0003727g0020* and *Nitab4.5_0003727g0010* expression in the alpha-linolenic acid metabolism (KO00592) pathways were significantly reduced in *HG14*, which was consistent with lower levels of linoleic acid and linolenic acid (**Figure 11**). The effect of gibberellins on fatty acid content has yet to be well studied. GA elevated the level of fatty acids through increased fatty acid desaturase expression by fermentation kinetics analysis in *Phaffia rhodozyma* (Liu et al., 2022). This was consistent with our discovery that the loss function of *NtGA3ox* resulted in decreased internal GA content. Exogenous GA, on the other hand, reduced linolenic acid levels in Canola (Elahi et al., 2023). GA3 was found to reduce the contents of linoleic and linolenic acid during early stages of seed filling by suppressing the formation of fatty acid desaturases FAD2 and FAD3 (Huang et al., 2014).

## 5 Conclusions

In conclusion, we identified a crucial gene designated *NtGA3ox1* that regulates tobacco growth and development. Knockout of *NtGA3ox1* resulted in impaired plant development, which extended flowering by 10 days and reduced morphological characteristics. The regulatory network of *NtGA3ox1* controlling tobacco flowering time were unveiled, plant-pathogen interaction, plant hormone signal transduction pathway, and MAPK signaling pathway were highly enriched. We established a correlation between gibberellin metabolism, glandular trichomes, and chemical component in tobacco. Further study is still required to fully understand how *NtGA3ox1* balances tobacco growth, stress resistance, and leaf quality.

## Data availability statement

The original contributions presented in the study are included in the article/**Supplementary Material**, further inquiries can be directed to the corresponding author/s.

## Author contributions

LD: Formal analysis, Investigation, Writing – original draft. CL: Formal analysis, Writing – original draft, Data curation. QG: Investigation, Writing – original draft. WY: Investigation, Writing – original draft. JJ: Investigation, Writing – original draft. JX: Investigation, Writing – original draft. HX: Formal analysis, Writing – original draft. JZ: Writing – review & editing. YY: Methodology, Writing – original draft, Conceptualization. PL: Funding acquisition, Project administration, Supervision, Writing – review & editing, Data curation.
