# Supplementary figures and images for: Loss function of NtGA3ox1 delays flowering through impairing gibberellins metabolite synthesis in Nicotiana tabacum
Source: Front Plant Sci. 2023 Dec 15;14:1340039. doi: 10.3389/fpls.2023.1340039 (PMC10754988; doi:10.3389/fpls.2023.1340039)

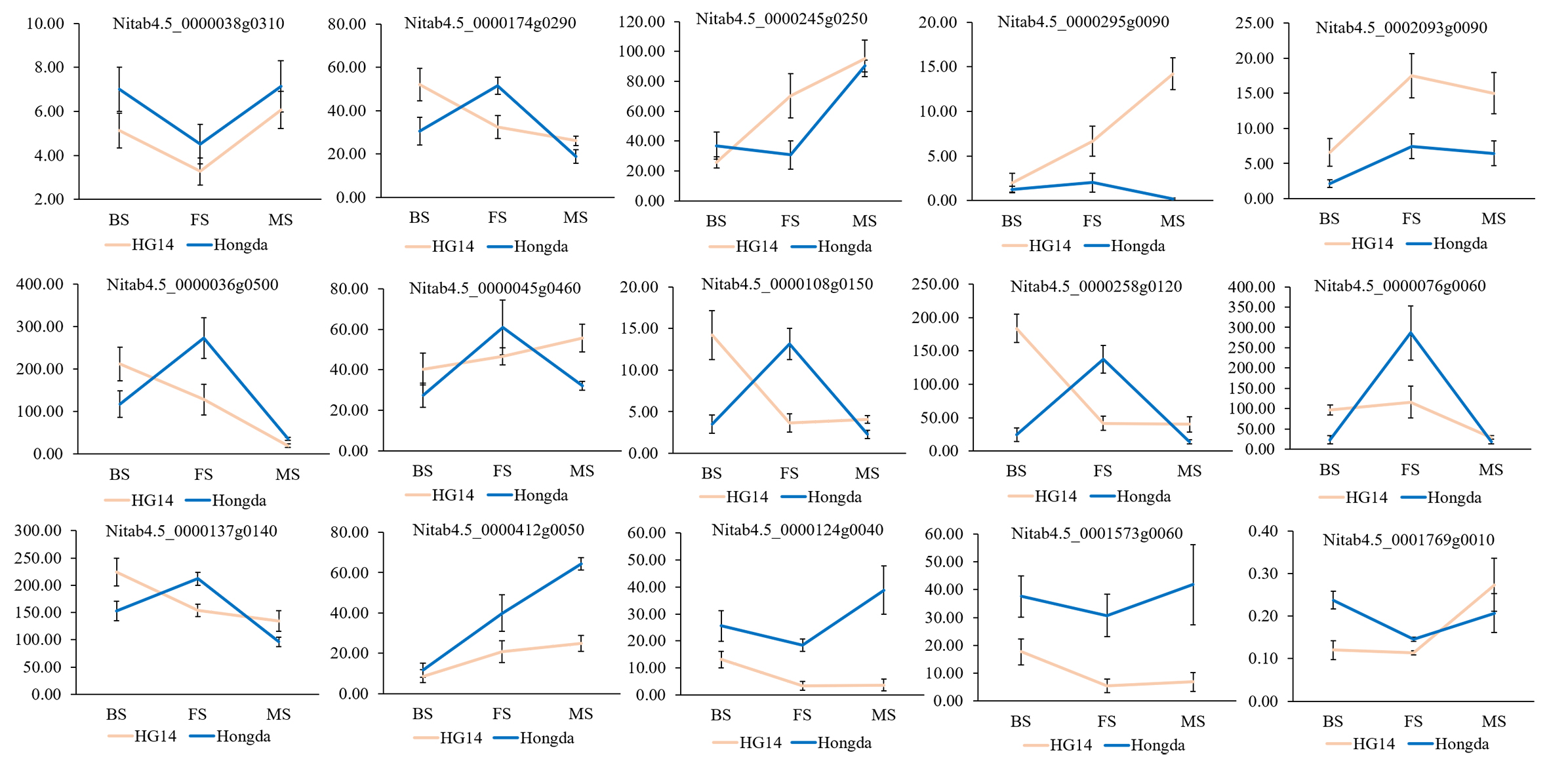

Supplement: Supplementary file 1 [file Image_1.jpeg]
