# Supplementary material for: Loss function of NtGA3ox1 delays flowering through impairing gibberellins metabolite synthesis in Nicotiana tabacum
Source: Front Plant Sci. 2023 Dec 15;14:1340039. doi: 10.3389/fpls.2023.1340039 (PMC10754988; doi:10.3389/fpls.2023.1340039)
Supplement: Supplementary file 3 [file Table_1.docx]

Supplementary Table 1 Primers used in this study.

| **Gene names** | **Primer names** | **Primer sequences (5'-3')** | **Purpose** |
| --- | --- | --- | --- |
| *NtGA3ox1* | P-GSP5 | ATTGGACCAAACCAAATTAACAATA | Mutation site identification |
|  | P-GSP3 | AGCTCCTTCCATATTATCCAGTAAA |  |
| *NtGA3ox1* | GA3-F | ATGCCTTCAAGAATCTCAGA | Gene cloning |
|  | GA3-R | TTAGCCAACTTGGACTCTATTA |  |
| *NtGA3ox1* | NtGA3ox-F1 | GATGCTTGGGTCACTTGGCA | Gene Expression analysis |
|  | NtGA3ox-R1 | GGCAGAGCATCCTCCTTTGG |  |
| *NtActin* | NtActin-F | GCATTGCTTGCTTTCACCCTT |  |
|  | NtActin-R | AACCTCCTTCACGATTTCATCATACC |  |
| *Nitab4.5_0000008g0310* | 310-F | GGTCTGCTTGGCCTGATTCT |  |
|  | 310-R | CGTGATATAGTGCCCGACCC |  |
| *Nitab4.5_0000174g0290* | 290-F | ATTTCACCCGAGGCATTCGT |  |
|  | 290-R | ATACACTGGCCACGAGCAAA |  |
| *Nitab4.5_0000245g0250* | 250-F | AAGCTACTGCCAACGAGTCC |  |
|  | 250-R | AGGAACTCTGCCAAGTGACG |  |
| *Nitab4.5_0000295g0090* | 590-F | CCATTCCAGTCGATGGGGTT |  |
|  | 590-R | AACAAGGCGACGACAAAACG |  |
| *Nitab4.5_0002093g0090* | 390-F | GCTTACAACATGCTACGCCG |  |
|  | 390-R | TCGCCCGATCAACAACATCA |  |
| *Nitab4.5_0000036g0500* | 500-F | CGGCGACGGTAAAATCTCCT |  |
|  | 500-R | CGTCGCCGTCAGTATCAACT |  |
| *Nitab4.5_0000045g0460* | 460-F | ATGAGCATCAACGTGGTCGT |  |
|  | 460-R | TGCAACACCTCTTCCTGCAT |  |
| *Nitab4.5_0000108g0150* | 150-F | CTCTACAGCACCACAAGGCA |  |
|  | 150-R | CATCCACACGACCTTTCCCA |  |
| *Nitab4.5_0000258g0120* | 120-F | ACAAGCACCTTTGTTGGGGA |  |
|  | 120-R | CGTCTCGCCTTCTCACTCTC |  |
| *Nitab4.5_0000076g0060* | 660-F | GATTCGGTGCTTCAATGGGC |  |
|  | 660-R | TCAGCCATTCTCCCTCCGTA |  |
| *Nitab4.5_0000137g0140* | 140-F | CTCGAGAAACTCGCTCTCCC |  |
|  | 140-R | CTTGACGGCGAGACTAGCAA |  |
| *Nitab4.5_0000412g0050* | 50-F | CGTCGTGAGGGAACTGGAAA |  |
|  | 50-R | TTTCACGGTGGTGATACCCG |  |
| *Nitab4.5_0000124g0040* | 40-F | CACGAGAACTTCCGGTTCCA |  |
|  | 40-R | GTAACGATGGGCGTTGGAGA |  |
| *Nitab4.5_0001573g0060* | 360-F | CGAGAACTTCCCGTTCCACT |  |
|  | 360-R | TGGGCGTTGCAGATGAGATT |  |
| *Nitab4.5_0001769g0010* | 10-F | GGGGCTTGTGGTGGAATGTA |  |
|  | 10-R | GGATCCACCAAGTGCCATGA |  |
