# Supplementary material for: Loss function of NtGA3ox1 delays flowering through impairing gibberellins metabolite synthesis in Nicotiana tabacum
Source: Front Plant Sci. 2023 Dec 15;14:1340039. doi: 10.3389/fpls.2023.1340039 (PMC10754988; doi:10.3389/fpls.2023.1340039)
Supplement: Supplementary file 4 [file Table_2.docx]

Supplementary Table 2 Transcriptome sequencing data quality processing results

| Sample | Total Raw Reads (M) | Total Clean Reads (M) | Total Clean Bases (Gb) | Clean Reads Q20 (%) | Clean Reads Q30 (%) | Clean Reads Ratio (%) |
| --- | --- | --- | --- | --- | --- | --- |
| MS-14_1 | 47.19 | 44.24 | 6.64 | 98.47 | 95.25 | 93.76 |
| MS-14_2 | 47.19 | 44.73 | 6.71 | 98.37 | 94.94 | 94.79 |
| MS-14_3 | 47.19 | 44.68 | 6.7 | 98.38 | 94.99 | 94.69 |
| MS-14_4 | 47.19 | 44.31 | 6.65 | 98.49 | 95.32 | 93.9 |
| MS-14_5 | 47.19 | 44.62 | 6.69 | 98.34 | 94.84 | 94.57 |
| MS-14_6 | 47.19 | 44.47 | 6.67 | 98.39 | 94.99 | 94.24 |
| MS-CK_1 | 47.19 | 44.7 | 6.7 | 98.44 | 95.12 | 94.73 |
| MS-CK_2 | 47.19 | 44.59 | 6.69 | 98.43 | 95.15 | 94.49 |
| MS-CK_3 | 47.19 | 44.24 | 6.64 | 98.41 | 95.09 | 93.76 |
| MS-CK_4 | 47.19 | 44.47 | 6.67 | 98.48 | 95.28 | 94.25 |
| MS-CK_5 | 48.93 | 44.92 | 6.74 | 98.45 | 95.2 | 91.8 |
| MS-CK_6 | 47.19 | 44.29 | 6.64 | 98.38 | 95 | 93.87 |
| FS-14_1 | 48.93 | 44.82 | 6.72 | 98.44 | 95.18 | 91.59 |
| FS-14_2 | 47.19 | 44.69 | 6.7 | 98.34 | 94.83 | 94.71 |
| FS-14_3 | 47.19 | 44.69 | 6.7 | 98.3 | 94.71 | 94.72 |
| FS-14_4 | 48.93 | 44.71 | 6.71 | 98.37 | 94.92 | 91.37 |
| FS-14_5 | 47.19 | 44.02 | 6.6 | 98.37 | 94.88 | 93.28 |
| FS-14_6 | 47.19 | 44.07 | 6.61 | 98.41 | 95.12 | 93.4 |
| FS-CK_1 | 47.19 | 44.61 | 6.69 | 98.41 | 95.09 | 94.54 |
| FS-CK_2 | 47.19 | 44.34 | 6.65 | 98.47 | 95.22 | 93.97 |
| FS-CK_3 | 46.61 | 43.08 | 6.46 | 98.39 | 95.12 | 92.42 |
| FS-CK_4 | 47.19 | 44.01 | 6.6 | 98.46 | 95.2 | 93.27 |
| FS-CK_5 | 48.93 | 45.35 | 6.8 | 98.47 | 95.25 | 92.68 |
| FS-CK_6 | 47.19 | 44.28 | 6.64 | 98.43 | 95.16 | 93.85 |
| BS-14_1 | 47.19 | 44.4 | 6.66 | 98.48 | 95.27 | 94.09 |
| BS-14_2 | 47.19 | 44.06 | 6.61 | 98.41 | 95.09 | 93.37 |
| BS-14_3 | 47.19 | 44.15 | 6.62 | 98.48 | 95.31 | 93.57 |
| BS-14_4 | 47.19 | 44.23 | 6.63 | 98.44 | 95.16 | 93.74 |
| BS-14_5 | 48.93 | 44.61 | 6.69 | 98.47 | 95.25 | 91.17 |
| BS-14_6 | 47.19 | 44.5 | 6.67 | 98.34 | 94.88 | 94.3 |
| BS-CK_1 | 47.19 | 43.85 | 6.58 | 98.34 | 94.91 | 92.92 |
| BS-CK_2 | 47.19 | 44.35 | 6.65 | 98.44 | 95.13 | 93.98 |
| BS-CK_3 | 48.93 | 44.67 | 6.7 | 98.63 | 95.73 | 91.3 |
| BS-CK_4 | 47.19 | 44.24 | 6.64 | 98.37 | 94.95 | 93.75 |
| BS-CK_5 | 47.19 | 44.58 | 6.69 | 98.11 | 94.07 | 94.47 |
| BS-CK_6 | 48.93 | 45.19 | 6.78 | 98.47 | 95.23 | 92.35 |
