# Supplementary material for: Loss function of NtGA3ox1 delays flowering through impairing gibberellins metabolite synthesis in Nicotiana tabacum
Source: Front Plant Sci. 2023 Dec 15;14:1340039. doi: 10.3389/fpls.2023.1340039 (PMC10754988; doi:10.3389/fpls.2023.1340039)
Supplement: Supplementary file 5 [file Table_3.docx]

Supplementary Table 3 18 compounds tested by Metabolome sequencing

| No. | Gibberellin |
| --- | --- |
| 1 | gibberellin A1 |
| 2 | gibberellin A15 |
| 3 | gibberellin A19 |
| 4 | gibberellin A20 |
| 5 | gibberellin A29 |
| 6 | gibberellin A3 |
| 7 | gibberellin A34 |
| 8 | gibberellin A4 |
| 9 | gibberellin A5 |
| 10 | gibberellin A51 |
| 11 | gibberellin A6 |
| 12 | gibberellin A7 |
| 13 | gibberellin A9 |
| 14 | gibberellin A24 |
| 15 | gibberellin A53 |
| 16 | gibberellin A8 |
| 17 | gibberellin A44 |
| 18 | gibberellin A12 aldehyde |
